# Protective Effect of Osmundacetone against Neurological Cell Death Caused by Oxidative Glutamate Toxicity

**DOI:** 10.3390/biom11020328

**Published:** 2021-02-22

**Authors:** Tuy An Trinh, Young Hye Seo, Sungyoul Choi, Jun Lee, Ki Sung Kang

**Affiliations:** 1 Saigon Pharmaceutical Science and Technology Center, University of Medicine and Pharmacy at Ho Chi Minh City, Ho Chi Minh 70000, Vietnam; tuyantrinh@gmail.com; 2 Herbal Medicine Resources Research Center, Korea Institute of Oriental Medicine (KIOM), Naju 58245, Korea; wnsl1118@kiom.re.kr; 3 College of Korean Medicine, Gachon University, Seongnam 13120, Korea; pc1075@gachon.ac.kr

**Keywords:** osmundacetone (OAC), *Elsholtzia ciliata*, oxidative stress, glutamate, apoptosis, MAPKs

## Abstract

Oxidative stress is one of the main causes of brain cell death in neurological disorders. The use of natural antioxidants to maintain redox homeostasis contributes to alleviating neurodegeneration. Glutamate is an excitatory neurotransmitter that plays a critical role in many brain functions. However, excessive glutamate release induces excitotoxicity and oxidative stress, leading to programmed cell death. Our study aimed to evaluate the effect of osmundacetone (OAC), isolated from *Elsholtzia ciliata* (Thunb.) Hylander, against glutamate-induced oxidative toxicity in HT22 hippocampal cells. The effect of OAC treatment on excess reactive oxygen species (ROS), intracellular calcium levels, chromatin condensation, apoptosis, and the expression level of oxidative stress-related proteins was evaluated. OAC showed a neuroprotective effect against glutamate toxicity at a concentration of 2 μM. By diminishing the accumulation of ROS, as well as stimulating the expression of heat shock protein 70 (HSP70) and heme oxygenase-1 (HO-1), OAC triggered the self-defense mechanism in neuronal cells. The anti-apoptotic effect of OAC was demonstrated through its inhibition of chromatin condensation, calcium accumulation, and reduction of apoptotic cells. OAC significantly suppressed the phosphorylation of mitogen-activated protein kinases (MAPKs), including c-Jun NH2-terminal kinase (JNK), extracellular signal-regulated kinase (ERK), and p38 kinases. Thus, OAC could be a potential agent for supportive treatment of neurodegenerative diseases.

## 1. Introduction

Neurological disorders are a major public health problem that constitutes more than 6% of the global burden of disease [1]. Among diseases of the central and peripheral nervous system, the most common neurological disorders are epilepsy, Alzheimer’s disease (AD), multiple sclerosis, cerebrovascular diseases, Parkinson’s disease (PD), and traumatic disorders. Approximately 45.9 million people worldwide were estimated to have epilepsy in 2016, and 1 in 10 individuals has suffered from at least one epileptic seizure in their lifetime [2]. Dementia was found to affect 47 million people globally in 2015, with AD contributing to 60–70% of cases [3]. The main pathogenesis mechanism of neurological disorders is the abnormality or death of brain cells caused by excitotoxic effects, disturbance of cellular energy metabolism, and oxidative stress [4].

Reactive oxygen species (ROS) or reactive nitrogen species (RNS) are the side products of aerobic respiration and cellular metabolism that act as regulatory mediators of physiological responses [5]. However, if the concentration of intracellular ROS or RNS exceeds the limitation of the antioxidant barrier of biological systems, it will cause oxidative stress, leading to apoptotic and necrotic cell death [6,7]. The brain consumes more oxygen than any other organ, which easily leads to excessive formation of ROS, but it is deficient in antioxidant capacity because of the ineffective activities of glutathione peroxidase (GPx) and catalase. Moreover, neuronal membranes contain large amounts of polyunsaturated fatty acids (PUFAs), which are highly susceptible to ROS during lipid peroxidation [8]. Consequently, the nervous system is especially vulnerable to the noxious effects of ROS as well as oxidative stress.

Oxidative stress is one of the major mechanisms that contributes to the development of neurological disorders. Mitochondrial dysfunction related to the excessive synthesis of ROS may lead to energy failure and apoptotic cell death in the early stage of neurodegeneration [9]. Dead neurons are commonly present in the brains of neurodegenerative patients, along with the typical morphological characteristics of apoptosis, such as DNA fragmentation, chromatin condensation, and caspase cascade activation [10]. Therefore, protecting the nervous system against oxidative stress is considered to be a potential target for clinical trials to develop drugs for the treatment of neurological disorders. Many antioxidants have been studied for their neuroprotective effects, with some positive initial results. Clinical trials in amyotrophic lateral sclerosis (ALS) patients showed that using vitamin E combined with riluzole for three months increased plasma GPx levels and diminished the levels of thiobarbituric acid reactive substances (TBARS) [11]. In another clinical trial by Arlt et al. [12], co-treatment with vitamin C and vitamin E during one year inhibited the cerebrospinal fluid (CSF) oxidation in AD patients. Some anti-Alzheimer drugs, such as donepezil, which increases the level of glutathione (GSH) [13], and memantine, which suppresses the plasma lipid oxidation rate in AD patients, have also shown their impact on oxidative stress biomarkers [14].

Glutamate is the major excitatory neurotransmitter in the mammalian brain [15], which plays an important role in learning and memory via its contribution to synaptic maintenance and plasticity [16]. However, glutamate is responsible for most of the oxidative stress in the central nervous system. The oxidative glutamate toxicity is induced by two main mechanisms: through the glutamate receptors or cystine transporter [17]. In the first mechanism, the binding of glutamate to its ionotropic receptor, the N-methyl-D-aspartate (NMDA) receptor, activates the peptidase calpain I, nitric oxide synthase, and phospholipase A2 that lead to the producing of ROS [18,19,20]. For the second mechanism, the interaction between glutamate and the cystine transporter results in the reduction of glutathione, an intracellular antioxidant, and the accumulation of ROS [21]. Glutamate neurotoxicity involves in not only acute injury, but also chronic neuronal degeneration such as Huntington’s disease, PD, multiple sclerosis, and amyotrophic lateral sclerosis [22,23]. Thus, glutamate is used to induce oxidative neuronal death in many cell lines models like HT22, PC12, or SH-SY5Y [24].

Natural compounds are an endless supply of material for the screening of antioxidants and neuroprotective agents. *Elsholtzia ciliata*, Thunb. Hylander, also known as Vietnamese balm, is widely distributed in Asian countries, and is commonly used as a vegetable, spice, herbal tea, and medicinal remedy. In folk and traditional medicine, *E. ciliata* is used for the treatment of colds, fever, abdominal pain, nausea, diarrhea, and asthma [25]. Moreover, many pharmacological effects of *E. ciliata* have been demonstrated in recent studies, such as antiviral [26], antioxidant [27,28], anti-inflammatory [28,29,30], anti-Leishmanial [31], and anti-acetylcholinesterase [32], as well as the upregulation of dopamine transporters [33]. From the known potential bioactivities of *E. ciliata*, we questioned whether osmundacetone (OAC), the compound isolated from this medicinal plant, has a neuroprotective effect against oxidative stress. This study aimed to evaluate the inhibitory effect of OAC on oxidative neurodegeneration and clarify the related mechanisms that contribute to its therapeutic effect. The model of glutamate-induced oxidative stress in HT22 murine hippocampal neuronal cells was used to investigate the efficacy of treatment with OAC on the ROS excess, intracellular calcium levels, chromatin condensation, and apoptosis. Western blot analysis was performed to study the regulatory effect of OAC on the phosphorylation of mitogen-activated protein kinases (MAPKs) and the expression of oxidative stress-related proteins.

## 2. Materials and Methods

### 2.1. Plant Material

Dried aerial parts of *E. ciliata* were purchased from KMD Medicinal Herbs Co. (Ulsan, Korea). This medicinal plant was authenticated by Dr. Choi Goya (Herbal Medicine Resources Research Center of the Korea Institute of Oriental Medicine (KIOM), Naju, Korea), and a voucher specimen, 2-20-0328, was deposited in the herbarium of the Herbal Medicine Resources Research Center of KIOM. The plant material (2.0 kg) was extracted by maceration in 70% ethanol for 48 h (three times) at room temperature and evaporated under reduced pressure. The resulting yield was 343.0 g of crude extract. Then this crude extract was dispersed in water and solvent-partitioned sequentially to obtain *n*-hexane, ethyl acetate (EtOAc), *n*-butanol, and water-soluble fractions.

### 2.2. Isolation of Compound

The EtOAc fraction (37.5 g) of *E. ciliata* was subjected to an Isolera One Spektra flash chromatography system (Biotage, Uppsala, Sweden) using a 340 g SNAP cartridge manually packed with Diaion HP-20 gel (Supelco, Bellefonte, PA, USA) with a gradient mobile phase of water/methanol (MeOH) from 100:0 to 0:100 to acquire 15 fractions (F01–15). Fraction F05 (1.2 g) was applied to a flash chromatography system using two 120 g SNAP Ultra C_18_ cartridges (water/MeOH, 100:0 to 40:60) to obtain 23 sub-fractions (F0501-23). The sub-fraction F0509 (47.5 mg) was separated into three 25 g SNAP cartridges manually packed with Sephadex LH-20 gel (MilliporeSigma, St. Louis, MO, USA) (water/MeOH, 100:0 to 60:40) followed by a 10 g SNAP Ultra cartridge (chloroform/MeOH/water, 95:5:0.5), using a flash chromatography system to obtain compound OAC (3.4 mg). OAC was identified as OAC by nuclear magnetic resonance (600 MHz, Agilent Technologies, Santa Clara, CA, USA) spectroscopic analysis.

### 2.3. Cell Culture

HT22 is a neuronal cell line derived from the mouse hippocampus [24]. HT22 cells were grown in Dulbecco’s modified Eagle’s medium (Corning, Manassas, VA, USA) and supplemented with 10% fetal bovine serum (Atlas Biologicals, Fort Collins, CO, USA), 100 units/mL penicillin, and 100 mg/mL streptomycin (Gibco, Grand Island, NY, USA). Cells were incubated at 37 °C in a humidified atmosphere under 5% CO_2_ and sub-cultured every two days.

### 2.4. Cell Viability Assay

HT22 cells were seeded in a 96-well plate at a density of 1 × 10^4^ cells/well. The next day, cells were co-treated with 5 mM glutamate and OAC at concentrations ranging from 0.5–4.0 μM to evaluate its protective effect against neuronal cell death. After 24 h of treatment, cell viability was determined by adding 10 μL EZ-Cytox assay reagent (DoGen, Seoul, Korea) in each well, incubating at 37 °C for 30 min, and then measuring the absorbance at 450 nm with a microplate reader (PowerWave XS; Bio-Tek Instruments, Winooski, VT, USA). The results of cell viability were presented as the relative value in percent compared with the control group (non-treated with glutamate and OAC; the cell viability is considered as 100%).

### 2.5. DPPH Radical Scavenging Assay

The antioxidant activity of OAC was evaluated using the 2,2-diphenyl-1-picrylhydrazyl (DPPH) assay. OAC in six concentrations (0–16 μM, including control) was tested by mixing with 60 μM DPPH (Sigma-Aldrich, St. Louis, MO, USA) at a ratio of 1:1; ethanol was used as the solvent. After 30 min at room temperature and protected from light, the absorbance of the reaction mixture was measured at 550 nm with a microplate reader (PowerWave XS; Bio-Tek Instruments, Winooski, VT, USA).

### 2.6. ROS Assay

HT22 cells were grown in 96-well plates for 24 h. The following day, cells were co-treated with 5 mM glutamate and OAC for 8 h. Next, cells were stained with 10 μM 2’,7’-dichlorodihydrofluorescein diacetate (H_2_DCF-DA; Sigma-Aldrich, St. Louis, MO, USA) in the culture medium for 30 min to detect ROS accumulation. After staining, cells were washed twice with Dulbecco’s phosphate-buffered saline (DPBS). The fluorescence intensity was measured using a fluorescence microplate reader (Spark 10M; Tecan, Männedorf, Switzerland), with excitation at 488 nm and emission at 525 nm. The fluorescence data were analyzed by SparkControl software. The fold increase was calculated based on the ratio of fluorescence intensity between the tested group and the control group (non-treated with glutamate and OAC; the fold increase is considered as 1).

### 2.7. Ca^2+^ Staining

HT22 cells were seeded in 24-well plates, and the next day, cells were co-treated with 5 mM glutamate and OAC for 8 h. Cells were then stained with 2.5 μM of fluo-4 AM (Invitrogen, Carlsbad, CA, USA) in the culture medium for 30 min to detect the accumulation of intracellular calcium. After staining, cells were washed twice with DPBS. The fluorescence intensity was measured using a fluorescence microplate reader (Spark 10M; Tecan, Männedorf, Switzerland), with excitation at 488 nm and emission at 525 nm. The fluorescence data were analyzed by SparkControl software. The fold increase was calculated based on the ratio of fluorescence intensity between the tested group and the control group (non-treated with glutamate and OAC; the fold increase is considered as 1).

### 2.8. Hoechst 33342 Staining

HT22 cells were cultured in six-well plates at a density of 2 × 10^5^ cells/well for 24 h. Afterward, cells were treated with OAC in the presence or absence of 5 mM glutamate for 12 h. Cells were stained with Hoechst 33342 (Sigma-Aldrich, Missouri, MO, USA) for 10 min, protected from light. Then, cells were washed with DPBS and observed the chromatin condensation under fluorescence microscopy (IX51; Olympus, Tokyo, Japan).

### 2.9. Image-Based Apoptosis Detection Assay

HT22 cells were grown in six-well plates at a density of 2 × 10^5^ cells/well and incubated for 24 h to adhere. The next day, cells were co-treated with 5 mM glutamate and OAC. After 12 h, cells were collected and washed in DPBS. Cells were stained with annexin V–Alexa Fluor 488 and propidium iodide (Invitrogen, Carlsbad, CA, USA) to determine the apoptotic or dead cells using a Tali Image-Based Cytometer (Invitrogen, Carlsbad, CA, USA), with the excitation wavelengths of 458 nm for green channel LED and 530 nm for red channel LED. The results obtained from the image-based apoptosis assay were analyzed by TaliPCApp.

### 2.10. Western Blotting Analysis

HT22 cells were seeded in six-well plates at a density of 2 × 10^5^ cells/well the day before. Then, cells were treated with OAC in the presence or absence of 5 mM glutamate. After 6 h, cells were collected and washed with DPBS, and lysed in RIPA buffer containing a 1X protease inhibitor cocktail to obtain whole-cell extracts. The protein concentration was determined using a Pierce BCA Protein Assay Kit (Thermo Scientific, Waltham, MA, USA). Equal amounts of protein in each sample (8 μg/lane) were electrophoresed in a 10% sodium dodecyl sulfate-polyacrylamide gel and transferred onto polyvinylidene difluoride (PVDF) membranes. Epitope-specific primary antibodies conjugated with secondary antibodies (Cell Signaling, Boston, MA, USA) were used to label the target proteins. The bound antibodies were detected with Pierce ECL Advance Western Blotting Detection Reagents (Thermo Scientific, Waltham, MA, USA) and visualized using a FUSION Solo Chemiluminescence System (PEQLAB Biotechnologie GmbH, Erlangen, Germany).

### 2.11. Statistical Analysis

All experiments were performed in triplicate for statistical analysis. The data are expressed as mean ± SEM. Statistical significance was determined using one-way ANOVA with Tukey HSD (honestly significant difference) post-hoc test. *P*-values of less than 0.001 or 0.005 were considered statistically significant.

## 3. Results

### 3.1. Neuroprotective Effect of OAC on HT22 Cells

**Figure 1:**
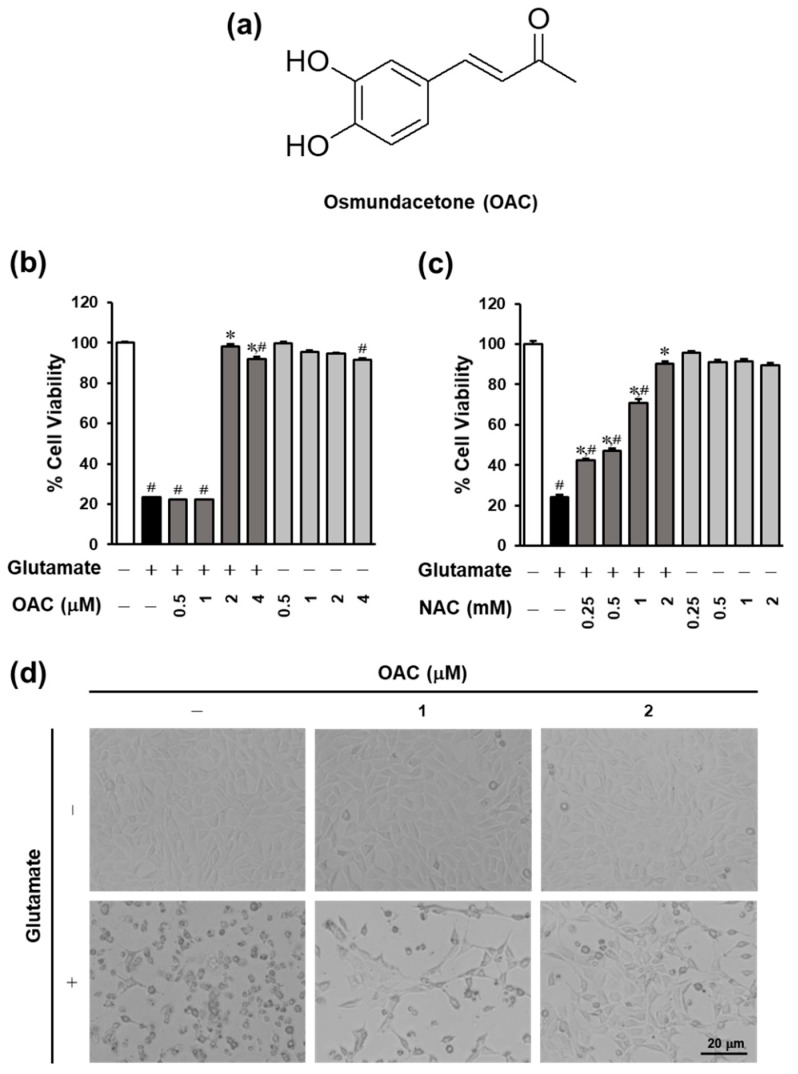
Neuroprotective effect of OAC against glutamate-induced HT22 hippocampal cell death. (**a**) The chemical structure of OAC. (**b**) Cell viability recovered by treatment with OAC in a dose-dependent manner. (**c**) N-acetylcysteine, used as a reference drug, prevented glutamate-induced HT22 cell death. (**d**) Cell morphology changes after 24 h of treatment with OAC at the concentration of 1 and 2 μM, in the presence or absence of 5 mM glutamate. OAC: osmundacetone; NAC: N-acetylcysteine; ^∗^ *p* < 0.001 vs. glutamate-treated group; ^#^ *p* < 0.001 vs. non-treated group.

The protective effect of OAC, isolated from *E. ciliata*, against glutamate-induced HT22 hippocampal cell death was evaluated using a cell viability assay. The results show that the cell vitality recovered to 98.26% ± 1.03% after treatment with OAC at the concentration of 2 μM (Figure 1). Meanwhile, treatment with N-acetylcysteine (NAC), which was used as a reference drug, restored the cell vitality to 90.39% ± 1.18% at the concentration of 2 mM. In Figure 1d, OAC also prevented the change of cell morphology caused by glutamate after 24 h. OAC shows the potential for neuroprotection.

### 3.2. Effect of OAC Against Oxidative Stress

**Figure 2:**
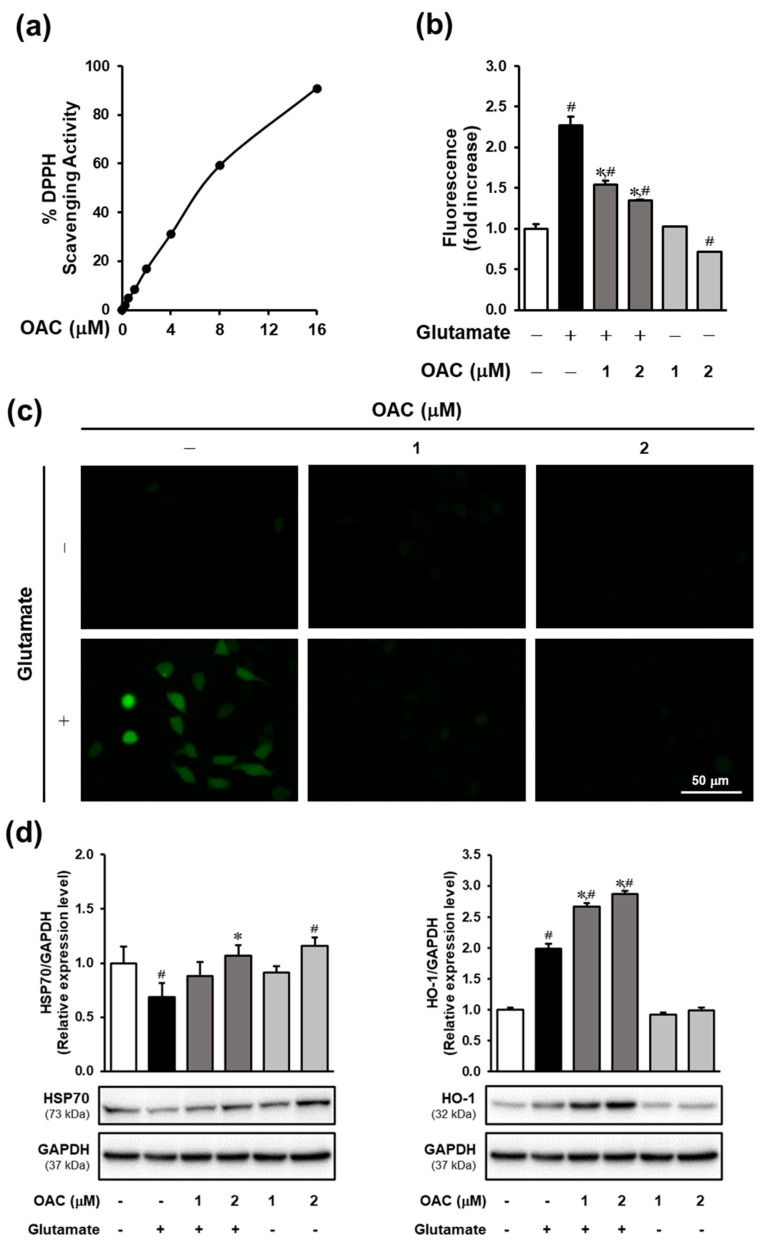
Effect of OAC against oxidative stress caused by the excess of glutamate in HT22 cells. (**a**) Antioxidant effect of OAC via DPPH scavenging activity; the IC_50_ was 7.88 ± 0.02 μM. (**b**) OAC significantly reduced the level of glutamate-induced ROS in a dose-dependent manner (^∗^ *p* < 0.005 vs. glutamate-treated group; ^#^ *p* < 0.005 vs. non-treated group). (**c**) Fluorescent images of H_2_DCF-DA staining. (**d**) Effect of OAC on the expression of oxidative stress-response enzymes (^∗^ *p* < 0.05 vs. glutamate-treated group; ^#^ *p* < 0.05 vs. non-treated group). GAPDH was used as a loading control. OAC: osmundacetone; DPPH: 2,2-diphenyl-1-picrylhydrazyl; ROS: reactive oxygen species; HSP70: heat shock protein 70; HO-1: heme oxygenase-1; GAPDH: glyceraldehyde-3-phosphate dehydrogenase.

The results of the DPPH assay showed that OAC had an antioxidant effect, with an IC_50_ of 7.88 ± 0.02 μM (Figure 2a). IC_50_ is defined here as the concentration giving half-maximal DPPH scavenging activity. The ROS assay was conducted to investigate the inhibitory effect of OAC on oxidative stress induced by glutamate in HT22 cells. As shown in Figure 2b, OAC significantly reduced the accumulation of ROS at both concentrations of 1 and 2 μM in a dose-dependent manner. The fluorescent images of H_2_DCF-DA staining also indicated that treatment with OAC suppressed the signal of glutamate-induced ROS (Figure 2c). The underlying mechanism of the reaction between OAC and oxidative stress-response enzymes was detected by Western blot analysis. After treatment with OAC, the protein expression of heat shock protein 70 (HSP70) and heme oxygenase-1 (HO-1) was induced in HT22 cells (Figure 2d).

### 3.3. Anti-Apoptotic Effect of OAC on HT22 Cells

**Figure 3:**
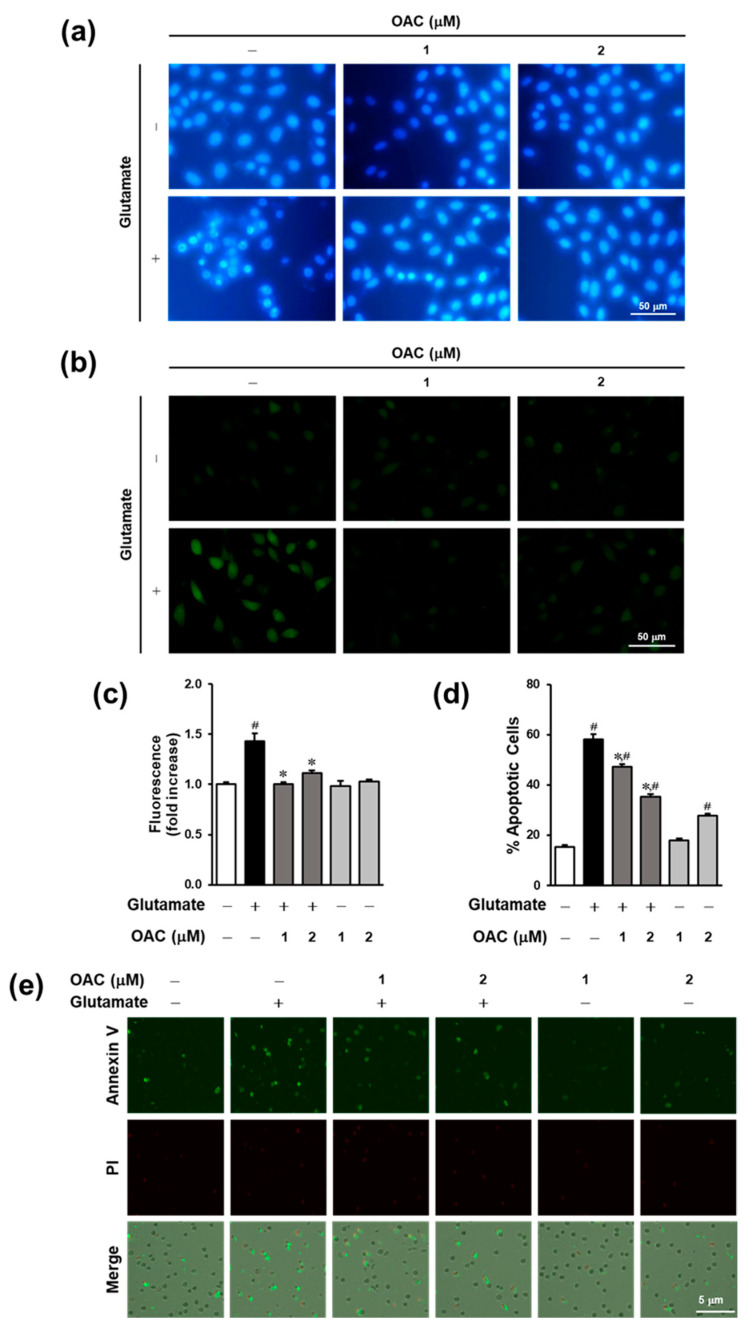
Anti-apoptotic effect of OAC against glutamate-induced neuronal cell death. (**a**) OAC prevented chromatin condensation in HT22 cells treated with glutamate. (**b**) Fluorescent images of fluo-4 staining. (**c**) OAC reduced the accumulation of intracellular calcium induced by glutamate in HT22 cells (^∗^ *p* < 0.005 vs. glutamate-treated group; ^#^ *p* < 0.005 vs. non-treated group). (**d**) The comparative graph illustrates the percentage of apoptotic cells (^∗^ *p* < 0.001 vs. glutamate-treated group; ^#^ *p* < 0.001 vs. non-treated group). (**e**) The microscopic images from an image-based apoptosis assay. OAC: osmundacetone; PI: propidium iodide.

The condensation of chromatin and accumulation of intracellular calcium are considered significant events during apoptosis. As shown in Figure 3a, Hoechst 33342 staining fluorescence microscopic images showed that OAC at a concentration of 2 μM prevented chromatin condensation in glutamate-induced HT22 cell death. The fluorescence intensity of Fluo-4, a Ca^2+^ indicator, was markedly reduced by treatment with OAC after 8 h (Figure 3b,c). Next, we performed an image-based apoptosis assay to confirm the anti-apoptotic effect of OAC. Apoptotic cells were labeled with annexin V–Alexa Fluor 488 dye, and the dead cells were detected by staining with propidium iodide. As shown in Figure 3d,e, the microscopic images and comparative graph show that glutamate sharply stimulated apoptosis to 58% ± 2.08%, whereas treatment with OAC at 1 and 2 µM diminished it to 47.33% ± 0.88% and 35.33% ± 0.88%, respectively.

### 3.4. Regulatory Effect of OAC on the Phosphorylation of MAPKs Induced by Glutamate

**Figure 4:**
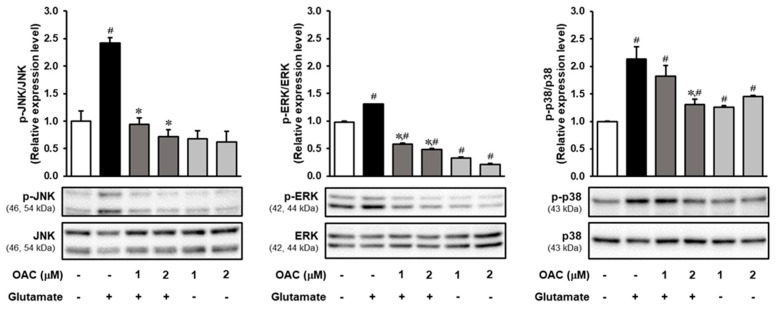
Inhibitory effect of OAC on the phosphorylation of MAPKs. OAC: osmundacetone; MAPKs: mitogen-activated protein kinases; JNK: c-Jun NH2-terminal kinase; ERK: extracellular signal-regulated kinase; ^∗^ *p* < 0.05 vs. glutamate-treated group; ^#^ *p* < 0.05 vs. non-treated group.

The results of Western blotting analysis, performed to investigate the effect of OAC on the phosphorylation of MAPKs, are presented in Figure 4. OAC markedly reduced the phosphorylation of c-Jun NH2-terminal kinase (JNK), which was stimulated by the excess of glutamate. The expression of phosphorylated p38 kinase was decreased by treatment with OAC at 2 μM. Compared to JNK and p38 kinase, glutamate induced the phosphorylation of extracellular signal-regulated kinase (ERK), and co-treatment with OAC downregulated its phosphorylation.

## 4. Discussion

OAC, also known as 4-(3,4-dihydroxyphenyl)-3-buten-2-one or 3,4-dihydroxybenzalacetone, was isolated from the EtOAc fraction of dried aerial parts of *E. ciliata*. Previous studies have reported several bioactivities of OAC, including antioxidant [34], anti-influenza [35], and anti-inflammation for attenuating acute lung injury in mice [36], as well as a protective effect against a gastric antral ulcer in rats [37] and the prevention of neuronal cell death [38,39]. OAC activates the Nrf2/glutathione pathway through PI3K/Akt [38], and induces oxidized protein-mediated endoplasmic reticulum stress and autophagy [39] to protect human neuroblastoma SH-SY5Y cells against neurotoxin 6-hydroxydopamine (6-OHDA) toxicity. These results suggest that OAC could be a promising neuroprotective agent. In order to widen the understanding of the active mechanisms related to the neuroprotective effect of OAC, we studied the role of this compound in alleviating oxidative glutamate toxicity in HT22 cells.

The HT22-immortalized hippocampal cell line is commonly used for the study of non-receptor mediated oxidative stress induced by glutamate. Although there is a lack of ionotropic glutamate receptors, high concentrations of glutamate (4–8 mM) still lead to HT22 cell death through the stimulation of ROS production [24]. Glutamate triggers oxidative stress in HT22 cells by suppressing the cystine uptake that leads to the depletion of intracellular antioxidant glutathione [40]. Next, oxidative stress activates the phosphorylation of JNK and p38, as well as the caspase independent pathway, which drives apoptosis [41]. Using the HT22 cell line allowed our study to focus on investigating the inhibitory effect of OAC on oxidative stress, with some predicted mechanistic pathways. The results show that OAC had a neuroprotective effect against glutamate toxicity that was more effective than NAC. NAC is a well-known antioxidant that induces GSH synthesis to protect cells from oxidative stress [42]. The efficacy of supportive treatment with NAC in neurological disorders such as PD, AD, and neuropathic pain has been proven by clinical trials [43].

Oxidative stress, which is induced by excess glutamate in neuronal cells, is characterized by the accumulation of intracellular free radicals [44]. OAC was identified as an antioxidant agent based on its DPPH scavenging activity, with an IC_50_ of 7.88 ± 0.02 μM. The role of OAC in preventing oxidative stress was confirmed by the reduced accumulation of ROS. Furthermore, OAC increased the expression of oxidative stress response enzymes, including HSP70 and HO-1. HSP70 is enhanced to protect neuronal cells from cerebral ischemia in animal models of neurodegenerative disease and trauma [45]. Oxidative stress results in the translocation of nuclear factor erythroid 2-related factor 2 (Nrf2) from the cytosol to the nucleus, which activates the antioxidant response element to initiate the transcription of cytoprotective genes like HO-1 [46]. In accordance with the previous study on the neuroprotective effect of OAC on SH-SY5Y cells, Gunjima et al. have mentioned that OAC stimulates the translocation of Nrf2, as well as inducing the expression of Nrf2-downstream genes like HO-1, cystine/glutamate antiporter (xCT), and glutamate-cysteine ligase modifier subunit (GCLM) [38].

The levels of both ROS and intracellular calcium (Ca^2+^) culminate in the last stage of oxidative glutamate toxicity that leads to apoptosis [40]. The accumulation of Ca^2+^ activates calpain and triggers apoptosis-inducing factor (AIF) translocation, which induces apoptosis, resulting in cell shrinkage, membrane blebbing, and chromatin condensation [41]. In our study, the level of intracellular Ca^2+^ was reduced by treatment with OAC, compared with the untreated group. OAC also inhibited chromatin condensation, a hallmark of apoptosis. An image-based apoptosis assay was performed to demonstrate the anti-apoptotic effect of OAC by decreasing the number of apoptotic cells.

**Figure 5:**
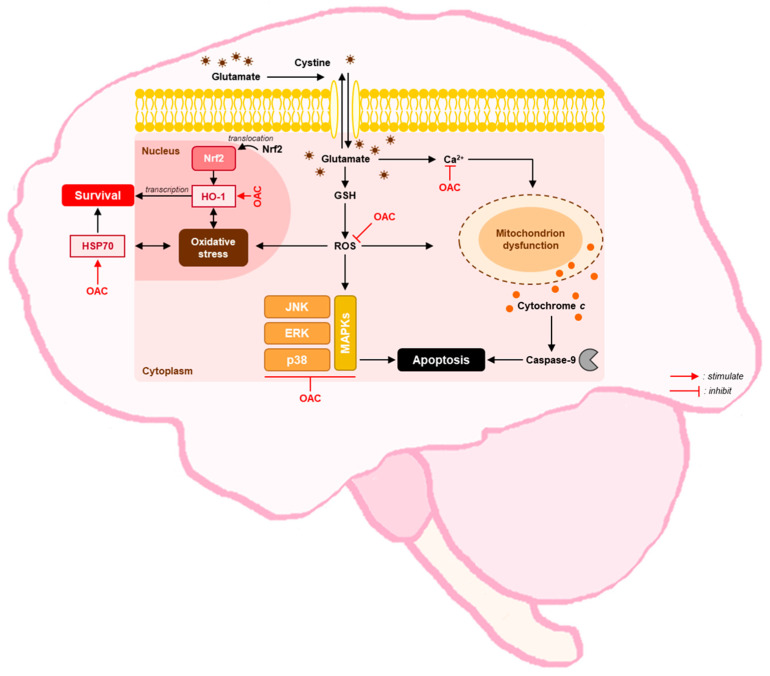
Protective mechanism of OAC against glutamate-induced oxidative stress in HT22 cells. OAC: osmundacetone; Ca^2+^: calcium ion; ERK: extracellular signal-regulated kinase; GSH: glutathione; HO-1: heme oxygenase-1; HSP70: heat shock protein 70; JNK: c-Jun NH2-terminal kinase; MAPKs: mitogen-activated protein kinases; Nrf2: nuclear factor erythroid 2-related factor 2; ROS: reactive oxygen species.

MAPKs play an important role in the regulation of metabolism, gene expression, and programmed cell death. The most popular subfamilies of MAPKs include JNKs, ERKs, and p38 kinases [47]. In AD, ROS can activate the expression of JNK and p38 under oxidative stress, driving Tau phosphorylation, which causes neuronal dysfunction and death [48]. Western blot analysis showed that OAC markedly reduced the phosphorylation of both JNK and p38 kinases. Furthermore, ROS stimulates the phosphorylation of ERK through the activation of epidermal growth factor (EGF) [49]. The phosphorylation of ERK was also suppressed after treatment with OAC. The protective mechanism of OAC against glutamate-induced oxidative stress is illustrated in Figure 5.

OAC suppressed the oxidative stress caused by glutamate, but OAC itself slightly stimulated both ROS and Ca^2+^ accumulation, as shown in Figure 2b and Figure 3c. Treatment with only OAC also increased the phosphorylation of p38 compared with the control group (Figure 4). These results revealed that from the concentration of 2 µM, OAC weakly induced oxidative stress in HT22 cells. The cytotoxicity of OAC should be investigated, and the lethal dose of OAC for animal experiments should be defined. Besides, in vitro studies on HT22 cells may not totally reflect the neuroprotective effect of OAC. In further studies, hippocampal primary culture and the related animal model are needed to support conclusions about the therapeutic effect of OAC for oxidative neurodegeneration.

## 5. Conclusions

In conclusion, OAC had a neuroprotective effect against oxidative glutamate toxicity in an HT22-immortalized hippocampal cell line. OAC reduced the accumulation of ROS, which prevented cellular oxidative stress. By stimulating the expression of HSP70 and HO-1, OAC triggered the self-defense mechanism of HT22 cells in response to excess exogenous glutamate. OAC also inhibited apoptosis, which is a consequence of oxidative stress. The hallmarks of apoptosis, such as chromatin condensation and excess of intracellular Ca^2+^, were suppressed by treatment with OAC. OAC reduced the phosphorylation of MAPKs, including JNK, ERK, and p38 kinases, which contributed to preventing neuronal cell death. These results suggested that OAC is a potential antioxidant agent that could be used for further in vivo studies and clinical trials to develop drugs for the treatment of neurological disorders.

## Data Availability

The data presented in this study are available on request from the cor-responding author.
